# New Neolignans and a Phenylpropanoid Glycoside from Twigs of *Miliusa mollis*

**DOI:** 10.3390/molecules15020639

**Published:** 2010-01-28

**Authors:** Kanokporn Sawasdee, Tanawat Chaowasku, Kittisak Likhitwitayawuid

**Affiliations:** 1 Department of Pharmacognosy, Faculty of Pharmaceutical Sciences, Chulalongkorn University, Bangkok 10330, Thailand; E-Mail: skanokporn7@hotmail.com; 2 Nationaal Herbarium Nederland, Leiden University, The Netherlands; E-Mail: chaowasku@nhn.leidenuniv.nl

**Keywords:** neolignan, phenylpropanoid glycoside, *Miliusa mollis*

## Abstract

From the twigs of *Miliusa mollis* Pierre, three new compounds including (2*S*,3*S*)-2,3-dihydro-2-(4-methoxyphenyl)-3-methyl-5-[1(E)-propenyl]benzofuran, (7*S*,8*S*)- *threo*-Δ^8′^-4-methoxyneolignan and tyrosol-1-*O*-*β*-xylopyranosyl-(1→6)-*O*-*β*-gluco-pyranoside were isolated, along with seven known compounds. Their structures were elucidated through analysis of their spectroscopic data.

## 1. Introduction

The genus *Miliusa* Lesch. ex A.DC. (Annonaceae) comprises 30–40 species, which occur from India and South China to North Australia [1]. So far, there have been only a few reports on the constituents of plants in this genus, describing the presence of aporphine alkaloids, terpenoids, flavonoids, phenylpropanoids, styrylpyrones, bis-styryls and homogentistic acid derivatives [2,3,4,5,6,7,8,9,10,11,12]. *Miliusa mollis* Pierre, is a shrub found in the northern and central regions of Thailand where it is locally known as Ching-chap [13]. Prior to this investigation, no studies had been done to examine the chemical components of this plant. The current paper describes the isolation and structural elucidation of three new compounds from the twigs of *M. mollis*.

## 2. Results and Discussion

**Figure 1:**
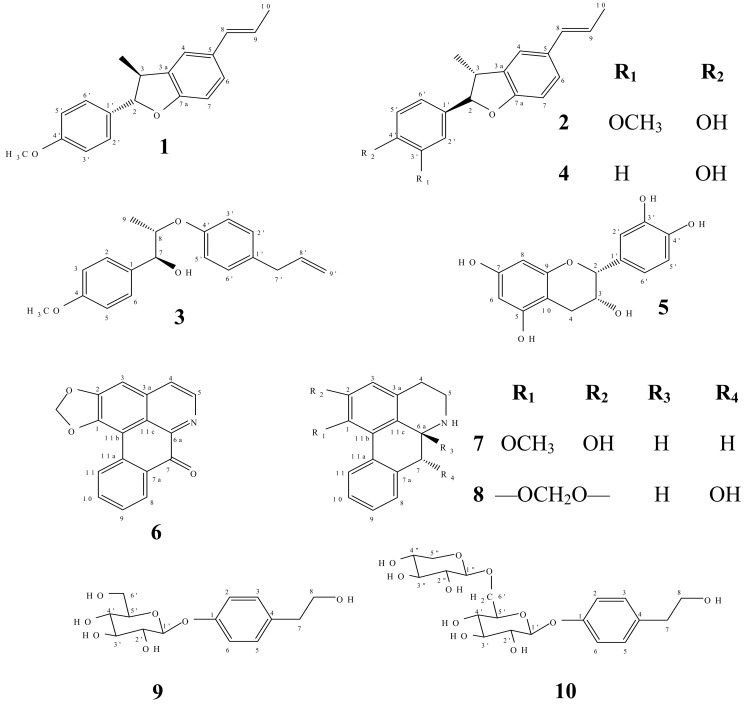
Compounds isolated from *Miliusa mollis.*

In this study, we report the isolation of two new neolignans including (2*S*,3*S*)-2,3-dihydro-2-(4-methoxyphenyl)-3-methyl-5-[1(E)-propenyl] benzofuran (**1**) and (7*S*,8*S*)- *threo*-Δ^8′^-4-methoxyneolignan (**3**), and a new glycosidic phenylpropanoid, namely tyrosol-1-*O*-*β*-xylopyranosyl-(1→6)-*O*-*β*-glucopyranoside (**10**), together with seven known compounds: (2*R*,3*R*)-2,3-dihydro-2-(4-hydroxy-3-methoxyphenyl)-3-methyl-5-(*E*)-propenylbenzofuran (**2**) [14], conocarpan (**4**) [14,15,16], (−)-epicatechin (**5**) [17,18], liriodenine (**6**) [19,20], asimilobine (**7**) [21,22], (−)-norushinsunine (**8**) [23] and icariside D_2_ (**9**) [24] (Figure 1). The structures of these known compounds were identified by comparison of their spectral data with those reported in the literature.

**Figure 2:**
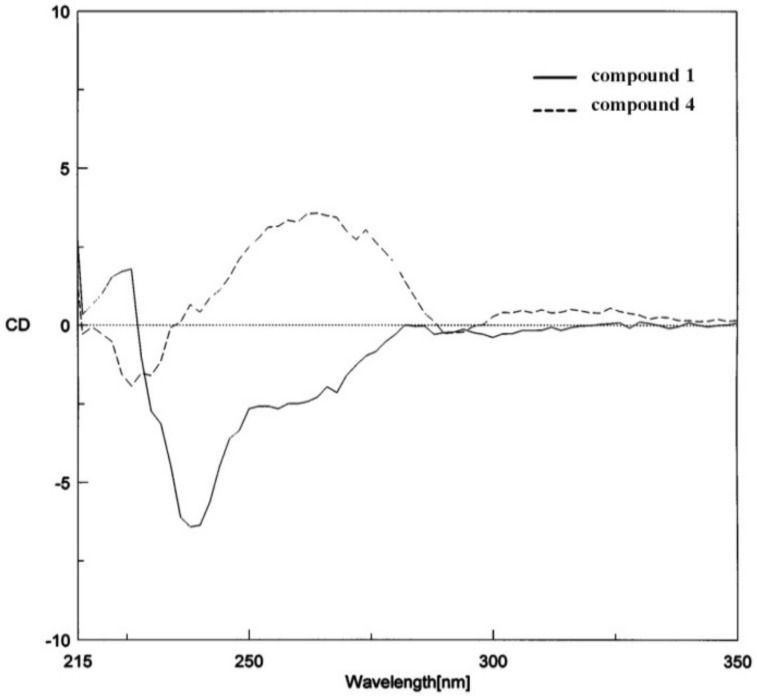
CD data of compounds **1** and **4**.

Compound **1** was obtained as a colorless oil. The positive HRESITOFMS exhibited an [M+Na]^+^ ion at *m/z* 303.1280, suggesting the molecular formula C_19_H_20_O_2_. The UV spectrum showed two absorption maxima at 228 and 274 nm, and the IR spectrum exhibited absorption bands for conjugated unsaturation (1,515 and 1,486 cm^−1^), and ether (1,243 cm^−1^) functionalities. The ^1^H-NMR signals at δ 5.09 (1H, d, *J* = 9.0 Hz, H-2), 3.39 (1H, m, H-3) and 1.39 (3H, d, *J* = 6.6 Hz, Me-3) and the ^13^C-NMR resonances at δ 92.6, 45.2, and 17.8 are characteristic features of the *trans*-2-aryl-3-methyl-2,3-dihydrobenzofuran system [14]. This was supported by the NOESY interactions of Me-3 protons with H-2. In the structure of **1**, a methoxy group [δ_H_ 3.81 (3H, s); δ_C_ 55.3] was present at C-4′, as indicated from the HMBC correlations from the protons at δ 3.81 to C-4′ (δ 158.3), and from H-2′(6′) (δ 7.35, 2H, d, *J* = 8.7 Hz) to C-2 (δ 92.6) and C-4′. In addition, a 2-propenyl moiety [δ_H_ 6.37 (1H, d, *J* = 15.8 Hz, H-8), 6.09 (1H, dq, *J* = 15.8, 6.3 Hz, H-9), 1.86 (3H, d, *J* = 6.3 Hz, Me-10); δ_C_ 130.8 (C-8), 122.9 (C-9), 18.3 (C-10)] was located at C-5 (δ 131.2), as evidenced by the ^3^*J*-coupling from C-5 to H-9 (δ 6.09). These spectral data appeared to be superimposable on those reported for synthetic (±)-*trans*-2,3-dihydro-2-(4-methoxyphenyl)-3-methyl-5-(1(*E*)-propenyl)benzofuran [25]. It is known that a *trans*-2-aryl-3-methyl-2,3-dihydrobenzofuran structure with 2*R*,3*R* configuration shows a positive Cotton effect at about 260 nm in the CD spectrum, whereas the reverse is true for the 2*S*,3*S*-isomer [14]. Since **1** showed a negative optical rotation $\left({\left[\alpha \right]}_{D}^{20}-13.22\right)$ and its CD curve exhibited a negative Cotton effect at 264 nm, the structure of **1** was determined as (2*S*,3*S*)-2,3-dihydro-2-(4-methoxyphenyl)-3-methyl-5-[1(E)-propenyl]benzofuran (Figure 1). Figure 2 shows the CD curve of **1**, in contrast with that of **4**, which is in the 2*R*,3*R* series. It should be noted that although the antipodal isomer of **1** was earlier mentioned [26,27], its spectroscopic data were not provided.

Compound **3** gave an [M+Na]^+^ ion at *m/z* 321.1375 in the HRESITOFMS, indicating a molecular formula of C_19_H_22_O_3_. The UV spectrum showed absorption maxima at 227 and 275 nm, and the IR spectrum demonstrated absorption bands for hydroxyl (3,448 cm^−1^), conjugated unsaturation (1,509 cm^−1^), and ether (1,243 cm^−1^) functionalities. The ^13^C-NMR spectrum of **3** (Table 1) showed a nineteen-carbon structure with two *p*-disubstituted benzene rings. In support of this, two pairs of doublets appeared at δ 7.32 (2H, d, *J* = 8.6 Hz, H-2 and H-6) and 6.88 (2H, d, *J* = 8.6 Hz, H-3 and H-5), and at δ 7.09 (2H, d, *J* = 8.4 Hz, H-2′ and H- 6′) and 6.87 (2H, d, *J* = 8.4 Hz, H-3′ and H-5′) in the ^1^H-NMR spectrum. In the HMQC spectrum, two tertiary oxygenated carbon signals appearing at δ 77.7 (C-7) and 79.3 (C-8) showed direct coupling with protons at δ 4.62 (1H, d, *J* = 7.7 Hz, H-7) and 4.34 (1H, dq, *J* = 7.7, 6.2 Hz, H-8), respectively. These two methine protons constituted an ABX coupling system with the Me protons at δ 1.07 (3H, d, *J* = 6.2 Hz, Me-9) in the COSY spectrum.

**Figure 3:**
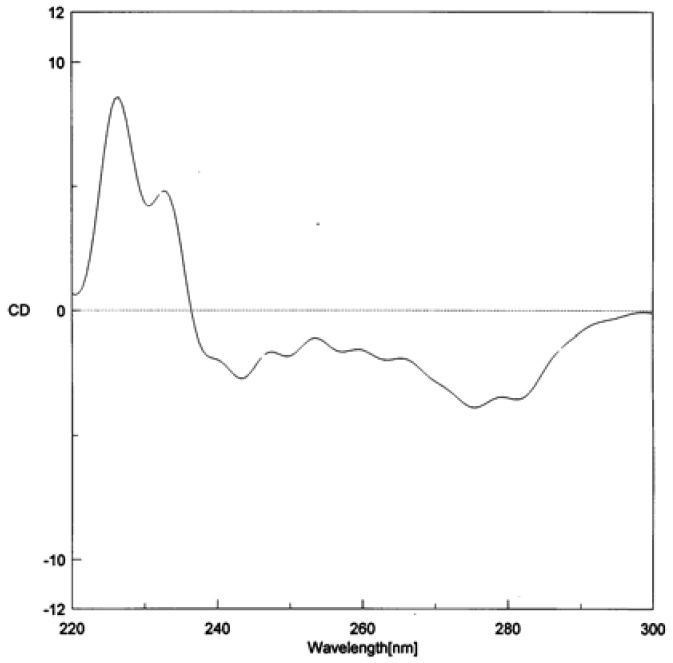
CD of data compound **3**.

Moreover, H-2 and H-6 exhibited 3-bond coupling with C-7, whereas H-8 showed HMBC connectivity to C-4′ through an ether linkage (Table 1). These spectral data of **3** were similar to those of previously reported 8-O-4′neolignans [28]. Compound **3** should have a methoxy group (δ_H_ 3.79, 3H, s; δ_C_ 55.3) at C-4 and an allyl moiety [(δ_H_ 3.32 (2H, br d, *J* = 6.6 Hz), 5.05 (2H, dd, *J* = 10.2, 16.8 Hz) and 5.93 (1H, m); δ_C_ 39.3, 115.5 and 137.7) at C-1′. The placement of the MeO group at C-4 was supported by the HMBC correlation from the MeO-4 protons (δ 3.79) to C-4 (159.6), which in turn showed ^3^*J*-coupling with H-2 and H-6. In accordance with this proposed structure, HMBC correlations were observed from C-1′ to H-3′(5′) and H-7′. It is known that for neolignans of this skeleton, the large coupling constant (*J* = 7.7 Hz) for H-7 and H-8, which was due to the intramolecular hydrogen bonding of the benzylic hydroxyl and the aryloxyl group, suggested a *threo* relative configuration [29,30]. On the basis of the negative and positive peaks at 276 and 233 nm, respectively in the CD spectrum (Figure 3), the absolute configurations at C-7 and C-8 of **3** were both assigned to be *S* [30]. Based on the above evidence, the structure of **3** was determined to be (7*S*,8*S*)-*threo*-Δ^8′^-4-methoxyneolignan.

Compound **10** was obtained as a colorless amorphous powder. It has a molecular formula of C_19_H_28_O_11_, as indicated by the [M+Na]^+^ ion peak at *m/z* 455.1619 in the HRESITOFMS. The compound showed UV absorptions at 223 and 273 nm, and IR bands at 3,366 (hydroxyl), 1,510 (conjugated unsaturation), and 1,071 and 1,043 (ether) cm^−1^. Compound **10** appeared to be a glycoside with tyrosol (4-hydroxyethylphenol) [31] as the aglycon, as suggested from the aromatic proton resonances at δ 7.10 (2H, d, *J* = 8.6 Hz, H-3 and H-5) and 6.95 (2H, d, *J* = 8.6 Hz, H-2 and H-6), and the aliphatic proton signals at δ 2.64 (2H, t, *J* = 6.5 Hz, H-7) and 3.54 (2H, t, *J* = 6.5 Hz, H-8) (Table 2). This was supported by the ^13^C-NMR signals at δ 155.7 (C-1), 132.7 (C-4), 129.7 (C-3 and C-5), and 116.2 (C-2 and C-6), 38.2 (C-7) and 62.4 (C-8) [31].

Apart from the tyrosol moiety, compound **10** possessed two sugar units, as evidenced by two anomeric protons at δ 4.73 (1H, d, *J* = 7.3 Hz, H-1′) and 4.17 (1H, d, *J* = 7.6 Hz, H-1″), which were correlated to the carbons at δ 100.7 (C-1′) and 103.8 (C-1″), respectively, in the HMQC spectrum. The inner sugar was *β*-glucopyranose [δ_H_ 4.73 (1H, d, *J* = 7.3 Hz, H-1′), 3.22 (2H, m, H-2′ and H-3′), 3.14 (1H, t, *J* = 8.8 Hz, H-4′), 3.48 (1H, dd, *J* = 8.8, 6.6 Hz, H-5′), 3.55 (1H, dd, 10.9, 6.6 Hz, H-6′_a_) and 3.93 (1H, dd, 10.9, 8.8 Hz, H-6′_b_); δ_C_ δ 100.7 (C-1′), 73.2 (C-2′), 76.5 (C-3′), 69.6 (C-4′), 75.8 (C-5′) and 68.2 (C-6′)] [32], and its connection to the aglycon through an arylether bond was demonstrated by the HMBC correlation from H-1′ to C-1 (δ 155.7) and the NOESY interaction of H-1′ with H-2(6). The other sugar unit was *β*-xylopyranose [δ_H_ 4.17 (1H, d, *J* = 7.6 Hz, H-1″), 2.96 (1H, dd, *J* = 8.7, 7.6 Hz, H-2″), 3.06 (1H, t, *J* = 8.7 Hz, H-3″), 3.22 (1H, m, H-4″), 3.65 (1H, dd, *J* = 11.3, 5.3 Hz, H-5″_b_), 2.94 (1H, t, *J* = 11.3 Hz, H-5″_a_); δ_C_ δ 103.8 (C-1″), 73.4 (C-2″), 76.5 (C-3″), 69.6 (C-4″), 65.6 (C-5″)], with its anomeric carbon linked to C-6′ of the glucose moiety through an ether bridge [30,33]. This linkage was further confirmed by the HMBC correlations between C-1″ and H_2_-6′, and between C-6′ and H-1″. Thus, the structure of **10** was determined to be tyrosol-1-*O*-*β*-xylopyranosyl-(1→6)-*O*-*β*-glucopyranoside.

It should be noted that although neolignans are frequently identified from the Annonaceae, they were not previously found in the genus *Miliusa*, and this is the first time that neolignans were isolated from a plant of this genus.

## 3. Experimental

### 3.1. General

Optical rotations were measured on a Perkin-Elmer 341 polarimeter, and the CD spectra were recorded on a JASCO J-715 spectropolarimeter. UV spectra were obtained on a Shimadzu UV-160A UV/vis spectrometer and IR spectra on a Perkin-Elmer FT-IR 1760X spectrophotometer. Mass spectra were recorded on a Micromass LCT mass spectrometer or a Thermo-Finnigan Polaris Q mass spectrometer. NMR spectra were obtained with a Bruker Avance DPX-300 FT-NMR spectrometer (300 MHz) or a JEOL JMN-A 500 NMR spectrometer (500 MHz). Vacuum-liquid column chromatography (VLC), column chromatography (CC) and medium pressure liquid chromatography (MPLC) were performed with silica gel 60 (Merck, Kieselgel 60, 70-230 mesh), silica gel 60 (Merck, Kieselgel 60, 230-400 mesh), Diaion HP20SS (Mitsubishi Chemical Co.) and Sephadex LH-20 (25–100 μm, Pharmacia Fine Chemical Co. Ltd.). Preparative TLC was carried out with silica gel plate (Merck, Kieselgel 60 F254).

### 3.2. Plant Material

The twigs of *Miliusa mollis* Pierre were collected in Bangkok, Thailand by one of us (T.C.) and identified by R. W. J. M. van der Ham, as previously described [1].

### 3.3. Extraction and Isolation

The dried and powdered plant material (380 g) was extracted with MeOH (3 × 3L) to give 24 g of an extract, which was then subjected to VLC on silica gel using solvent mixtures of increasing polarity (*n*-hexane, CH_2_Cl_2_, EtOAc and MeOH) to give eight fractions (A-H).

Fraction D (98 mg) was further separated by CC on silica gel (15.4 g) with gradient elution (*n*-hexane-CH_2_Cl_2_) to give seven fractions (D1–D7). Fraction D4 (33 mg) was purified on Sephadex LH-20 (CH_2_Cl_2_-MeOH 1:1) to give **1** (22 mg).

Fraction E (321 mg) was separated by CC on silica gel (21.62 g) with *n*-hexane-CH_2_Cl_2_ gradient elution to give ten fractions (E1-E10). Fraction E6 (184 mg) was separated on Sephadex LH-20 (CH_2_Cl_2_-MeOH 1:1) to give **2** (100 mg).

Fraction F (1.9 g) was separated by MPLC (silica gel, *n*-hexane-CH_2_Cl_2_ gradient elution) to give eleven fractions (F1–F11). Fraction F7 (171.9 mg) was separated with Sephadex-LH-20/CH_2_Cl_2_-MeOH (1:1) to give six fractions (F7-1 to F7-6). Fraction F7-4 (7 mg) was purified by preparative TLC (silica gel, *n*-hexane-EtOAc-acetone 90:8:2) to yield 2 mg of **3**. Fraction F8 (379 mg) was separated with Sephadex LH-20/CH_2_Cl_2_-MeOH (1:1) to give **4** (339.0 mg).

Fraction G (1.6 g) was separated by MPLC (silica gel, *n*-hexane-EtOAc gradient elution) to give twelve fractions (G1–G12). Fraction G9 (433.4 mg) was separated on Sephadex LH-20 (acetone) to give **5** (163 mg).

Fraction H (16.5 g) was fractionated on Diaion HP20SS, eluted with H_2_O-MeOH (100:0–0:100) to give seven fractions (H1-H7). Fraction H7 (405.9 mg) was separated on silica gel (21.0 g) with EtOAc-MeOH-H_2_O gradient elution to give fourteen fractions (H7-1 to H7-14). Fraction H7-2 (25 mg) was further purified with Sephadex-LH-20/CH_2_Cl_2_-MeOH (1:1) to give **6** (2 mg). Fraction H7-6 (52 mg) was separated on Sephadex LH-20, eluted with CH_2_Cl_2_-MeOH (1:1) to give four fractions (H7-6-1 to H7-6-4). Fraction H7-6-4 (10 mg) was further purified by CC (silica gel, CH_2_Cl_2_-acetone 1:4) to give **7** (2 mg). Fraction H7-7 (38 mg) was separated with Sephadex LH-20/CH_2_Cl_2_-MeOH (1:1) to give four fractions (H7-7-1 to H7-7-4). Fraction H7-7-4 (15 mg) was further purified by CC on silica gel, eluted with CH_2_Cl_2_-acetone (1:4) to give 2 mg of **7** and 5 mg of **8**. Fraction H4 (282 mg) was separated by CC on silica gel (19.6 g) with EtOAC-MeOH-H_2_O gradient elution to give twelve fractions (H4-1 to H4-12). Fraction H4-3 (16 mg) was separated on Sephaddex LH-20(MeOH) to give three fractions (H4-3-1 to H4-3-3). Fraction H4-3-1 (14 mg) was further purified by preparative TLC (silica gel) with EtOAc-MeOH-H_2_O (92:6:2) to yield 10 mg of **9**. Fraction H4-6 (39 mg) was separated on Sephadex LH-20 (MeOH) to give six fractions (H4-6-1 to H4-6-6). Fraction H4-6-4 (26 mg) was further purified by CC (silica gel, EtOAc-MeOH-H_2_O 80:12:8) to give **10** (3 mg).

*(2S,3S)-2,3-Dihydro-2-(4-methoxyphenyl)-3-methyl-5-*[1(E)-propenyl]*benzofuran* (**1**): colorless oil; ${\left[\alpha \right]}_{D}^{20}-13.22$ (*c* 0.42, MeOH); CD (MeOH, *c* 0.001): [θ]_300_ −401, [θ]_264_ −2,296, [θ]226 +1,791; EI-MS *m/z* 281 [M+1]^+^, 280 [M]^+^, 265, 251, 157, 148, 135, 131, 115, 103, 91; HRESITOFMS *m/z* 303.1280 [M+Na]^+^ (calcd. for C_19_H_20_O_2_Na, 303.1361); UV λ_max_ (MeOH) nm (log ε) 228 (3.76), 274 (3.26); IR ν_max_ (film): 1515, 1486, 1243 cm^-1^; ^1^H-NMR (300 MHz, CDCl_3_): δ 7.35 (2H, d, *J* = 8.7 Hz, H-2′, H-6′), 7.14 (1H, br s, H-4), 7.12 (1H, d, *J* = 8.1 Hz, H-6), 6.91 (2H, d, *J* = 8.7 Hz, H-3′, H-5′), 6.76 (1H, d, *J* = 8.1 Hz, H-7), 6.37 (1H, d, *J* = 15.8 Hz, H-8), 6.09 (1H, dq, *J* = 15.8, 6.3 Hz, H-9), 5.09 (1H, d, *J* = 9.0 Hz, H-2), 3.81 (3H, s, MeO), 3.39 (1H, m, H-3), 1.86 (3H, d, *J* = 6.3 Hz, Me-10), 1.39 (3H, d, *J* = 6.6 Hz, Me-3); ^13^C-NMR (75 MHz, CDCl_3_): δ 159.6 (C-7a), 158.3 (C-4′), 132.7 (C-3a), 132.4 (C-1′), 131.2 (C-5), 130.8 (C-8), 127.6 (C-2′, C-6′), 126.3 (C-6), 122.9 (C-9), 120.7 (C-4), 114.0 (C-3′,C-5′), 109.2 (C-7), 92.6 (C-2), 45.2 (C-3), 55.3 (MeO), 18.3 (C-10), 17.8 (Me-3).

*(7S,8S)-threo-Δ^8^^′^-4-methoxyneolignan* (**3**): colorless oil; ${\left[\alpha \right]}_{D}^{20}+10.0$ (*c* 0.05, MeOH); CD (MeOH, *c* 0.002): [θ]_276_ −1,932, [θ]_233_ +2,392; EI-MS *m/z* 298 [M]^+^, 281, 162, 161, 137, 133, 121, 115, 105, 91, 77; HRESITOFMS *m/z* 321.1375 [M+Na]^+^ (calcd. for C_19_H_22_O_3_Na, 321.1468); UV λ_max_ (MeOH) nm (log ε) 227 (4.18), 275 (3.48); IR ν_max_ (film): 3448 (br), 1509, 1243 cm^-1^; ^1^H-NMR (300 MHz, CDCl_3_) and ^13^C-NMR (75 MHz, CDCl_3_): see Table 1.

*Tyrosol-1-O-β-xylopyranosyl-(1→6)-O-β-glucopyranoside* (**10**): colorless amorphous powder; ${\left[\alpha \right]}_{D}^{20}-48.75$ (*c* 0.08, MeOH); EI-MS *m/z* 414, 207, 167, 149, 138, 107, 77; HRESITOFMS *m/z* 455.1619 [M+Na]^+^ (calcd. for C_19_H_28_O_11_Na, 455.1529); UV λ_max_ (MeOH) nm (log ε) 223 (3.51), 273 (2.77); IR ν_max_ (film): 3366 (br), 1510, 1071, 1043 cm^-1^; ^1^H-NMR (500 MHz, DMSO-*d*_6_) and ^13^C-NMR (125 MHz, DMSO-*d*_6_): see Table 2.

## 4. Conclusion

Three new compounds including (2*S*,3*S*)-2,3-dihydro-2-(4-methoxyphenyl)-3-methyl-5-[1(E)-propenyl]benzofuran, (7*S*,8*S*)- *threo*-Δ^8′^-4-methoxyneolignan and tyrosol-1-*O*-*β*-xylopyranosyl-(1→6)-*O*-*β*-glucopyranoside were isolated from the twigs of *Miliusa mollis* Pierre. The presence of neolignans in the genus *Miliusa* was reported for the first time in this study.

## Figures and Tables

**Table 1:** ^1^H- (300 MHz) and ^13^C-NMR (75 MHz) data of **3** (CDCl_3_, δ in ppm and *J* in Hz) and HMBC correlations.

Position	^1^H	^13^C	HMBC (correlation with ^1^H)
1	-	132.0 (s)	3, 5 and 7
2	7.32 (1H, d, 8.6)	128.5 (d)	6 and 7
3	6.88 (1H, d, 8.6)	113.9 (d)	5
4	-	159.6 (s)	2, 6 and MeO
5	6.88 (1H, d, 8.6)	113.9 (d)	3
6	7.32 (1H, d, 8.6)	128.5 (d)	2 and 7
7	4.62 (1H, d, 7.7)	77.7 (d)	8 and 9
8	4.34 (1H, dq, 7.7, 6.2)	79.3 (d)	9
9	1.07 (3H, d, 6.2)	15.7 (q)	
1′	-	133.1 (s)	3′, 5′ and 7′
2′	7.09 (1H, d, 8.4)	129.7 (d)	6′ and 7′
3′	6.87 (1H,d, 8.4)	116.4 (d)	
4′	-	156.1 (s)	8, 2′ and 6′
5′	6.87 (1H,d, 8.4)	116.4 (d)	
6′	7.09 (1H, d, 8.4)	129.7 (d)	2′ and 7′
7′	3.32 (2H, br d, 6.6)	39.3 (t)	
8′	5.93 (1H, m)	137.7 (d)	7′
9′	5.05 (2H, dd, 10.2, 16.8)	115.5 (t)	7′
MeO-4	3.79 (3H, s)	55.3 (q)	-

**Table 2:** ^1^H- (500 MHz) and ^13^C-NMR (125 MHz) data of **10** (DMSO-*d*_6_, δ in ppm and *J* in Hz) and HMBC correlations.

Position	^1^H	^13^C	HMBC (correlation with ^1^H)
1	-	155.7 (s)	2, 3, 5, 6 and 1′
2	6.95 (1H, d, 8.6)	116.2 (d)	3 and 6
3	7.10 (1H, d, 8.6)	129.7 (d)	2, 5 and 7
4	-	132.7 (s)	2, 6, 7 and 8
5	7.10 (1H, d, 8.6)	129.7 (d)	3, 6 and 7
6	6.95 (1H, d, 8.6)	116.2 (d)	2 and 5
7	2.64 (2H, t, 6.5)	38.2 (t)	3, 5 and 8
8	3.54 (2H, t, 6.5)	62.4 (t)	7
1′	4.73 (1H, d, 7.3)	100.7 (d)	5′
2′	3.22 (1H, m)	73.2 (d)	3′
3′	3.22 (1H, m)	76.5 (d)	1′
4′	3.14 (1H, t, 8.8)	69.6 (d)	2′,3′, 5′ and 6′_b_
5′	3.48 (1H, dd, 8.8, 6.6)	75.8 (d)	1′ and 6′_a_
6′_a_	3.55 (1H, dd, 10.9, 6.6)	68.2 (t)	-
6′_b_	3.93 (1H, dd, 10.9, 8.8)	-	5′ and 1″
1˝	4.17 (1H, d, 7.6)	103.8 (d)	5″_a_ and 5″_b_, 6′_a_, 6′_b_
2˝	2.96 (1H, dd, 8.7, 7.6)	73.4 (d)	1″ and 3″
3˝	3.06 (1H, t, 8.7)	76.5 (d)	2″, 5″_a_ and 5″_b_
4˝	3.22 (1H, m)	69.6 (d)	2″, 3″, 5″_a_ and 5″_b_
5˝_a_	2.94 (1H, t, 11.3)	65.6 (t)	-
5˝_b_	3.65 (1H, dd, 11.3, 5.3)	-	1″
